# The number of tracheal intubation attempts matters! A prospective multi-institutional pediatric observational study

**DOI:** 10.1186/s12887-016-0593-y

**Published:** 2016-04-29

**Authors:** Jan Hau Lee, David A. Turner, Pradip Kamat, Sholeen Nett, Justine Shults, Vinay M. Nadkarni, Akira Nishisaki, for the Pediatric Acute Lung Injury and Sepsis Investigators (PALISI), the National Emergency Airway Registry for Children (NEAR4KIDS)

**Affiliations:** Children’s Intensive Care Unit, KK Women’s and Children’s Hospital, 100 Bukit Timah Road, Singapore, 229899 Singapore; Duke-NUS Medical School, Singapore, Singapore; Division of Pediatric Critical Care Medicine, Department of Pediatrics, Duke Children’s Hospital, Durham, NC USA; Department of Pediatrics, Emory University School of Medicine, Atlanta, GA USA; Critical Care Medicine, Children’s Healthcare of Atlanta at Egleston, Atlanta, GA USA; Division of Pediatric Critical Care, Dartmouth-Hitchcock Medical Center, Lebanon, NH USA; Department of Biostatistics and Epidemiology, University of Pennsylvania Perelman School of Medicine, Philadelphia, USA; Department of Anesthesiology and Critical Care Medicine, The Children’s Hospital of Philadelphia, Philadelphia, PA USA

**Keywords:** Acute respiratory failure, Child, Critical illness, Desaturation, Intubation, Mechanical ventilation, Pediatric intensive care unit, Registries

## Abstract

**Background:**

The impact of multiple tracheal intubation (TI) attempts on outcomes in critically ill children with acute respiratory failure is not known. The objective of our study is to determine the association between number of TI attempts and severe desaturation (S_p_O_2_ < 70 %) and adverse TI associated events (TIAEs).

**Methods:**

We performed an analysis of a prospective multicenter TI database (National Emergency Airway Registry for Children: NEAR4KIDS). Primary exposure variable was number of TI attempts trichotomized as one, two, or ≥3 attempts. Estimates were adjusted for history of difficult airway, upper airway obstruction, and age. We included all children with initial TI performed with direct laryngoscopy for acute respiratory failure between 7/2010-3/2013. Our main outcome measures were desaturation (<80 % during TI attempt), severe desaturation (<70 %), adverse and severe TIAEs (e.g., cardiac arrest, hypotension requiring treatment).

**Results:**

Of 3382 TIs, 2080(65 %) were for acute respiratory failure. First attempt success was achieved in 1256/2080(60 %), second attempt in 503/2080(24 %), and ≥3 attempts in 321/2080(15 %). Higher number of attempts was associated with younger age, history of difficult airway, signs of upper airway obstruction, and first provider training level. The proportion of TIs with desaturation increased with increasing number of attempts (1 attempt:16 %, 2 attempts:36 %, ≥3 attempts:56 %, *p* < 0.001; adjusted OR for 2 attempts: 2.9[95 % CI:2.3–3.7]; ≥3 attempts: 6.5[95 % CI: 5.0–8.5], adjusted for patient factors). Proportion of TIs with severe desaturation also increased with increasing number of attempts (1 attempt:12 %, 2 attempts:30 %, ≥3 attempts:44 %, *p* < 0.001); adjusted OR for 2 attempts: 3.1[95 % CI:2.4–4.0]; ≥3 attempts: 5.7[95 % CI: 4.3–7.5] ). TIAE rates increased from 10 to 29 to 38 % with increasing number of attempts (*p* < 0.001); adjusted OR for 2 attempts: 3.7[95 % CI:2.9–4.9] ; ≥3 attempts: 5.5[95 % CI: 4.1–7.4]. Severe TIAE rates went from 5 to 8 to 9 % (*p* = 0.008); adjusted OR for 2 attempts: 1.6 [95 % CI:1.1–2.4]; ≥3 attempts: 1.8[95 % CI:1.1–2.8].

**Conclusions:**

Number of TI attempts was associated with desaturations and increased occurrence of TIAEs in critically ill children with acute respiratory failure. Thoughtful attention to initial provider as well as optimal setting/preparation is important to maximize the chance for first attempt success and to avoid desaturation.

**Electronic supplementary material:**

The online version of this article (doi:10.1186/s12887-016-0593-y) contains supplementary material, which is available to authorized users.

## Background

The need for tracheal intubation (TI) and mechanical ventilation (MV) is one of the most common indications for admission to pediatric intensive care units (PICUs), with a significant proportion of children with acute respiratory failure in PICUs requiring TI and invasive mechanical ventilation [1–3]. Critically ill children in the PICU have decreased physiologic reserve due to hemodynamic and respiratory decompensation, and the urgent situations necessitating TI put children at increased risk for adverse events during intubations [4]. In addition to the increased risk related to critical illness, data from the adult literature demonstrate that multiple attempts to achieve TI are associated with increased risk for TI associated clinical deterioration in both intensive care units (ICUs) and emergency departments [5–7].

In children, there are very few reports addressing the association between multiple TI attempts and adverse clinical outcomes, with previous investigations being mainly performed in clinical settings outside the PICUs (e.g., emergency departments, neonatal ICUs) [8–11]. Prior studies that specifically examine the association between number of TI attempts and clinical outcomes in critically ill children in PICUs are limited to single center investigations. Those studies did not specifically examine the association of multiple TI attempts with outcomes in patients with acute respiratory failure [4, 12]. We focused on acute respiratory failure because this diagnostic category constitutes a significant proportion of children admitted to the PICU, and these patients likely have a higher risk profile than many other populations of patients who require TI and MV. Furthermore, an overwhelming majority of children with acute respiratory failure require TI and MV [2].

Our objective of this study was to evaluate the association of multiple TI attempts with immediate safety outcomes, accounting for patient characteristics previously associated with adverse events, using a multicenter TI quality improvement database: the National Emergency Airway Registry for Children (NEAR4KIDS). We hypothesized that an increased number of TI attempts would be associated with increased occurrence of desaturation [pulse oximetry (SpO_2_ < 80 %)], severe desaturation (SpO_2_ < 70 %), and with adverse TI associated events (TIAEs) in children who are intubated for acute respiratory failure in the PICU.

## Methods

The National Emergency Airway Registry for Children (NEAR4KIDS) is a prospective multicenter TI collaborative that, at the time of this study, included 19 PICUs worldwide (15 in the United States, 1 in Canada, 1 in Japan, 1 in Singapore, and 1 in New Zealand). The Institutional Review Board at the Children’s Hospital of Philadelphia approved the multicenter study protocol. In addition, all participating sites and the data coordinating center received an approval from respective institutional review board (IRB). All IRBs granted a waiver of consent. Participating sites are listed in Additional file 1. Each center developed a data compliance plan to ensure that more than 95 % TIs were captured in a timely fashion, reconciled, and entered in the database [12]. The Compliance Officer for NEAR4KIDS reviewed and approved the plan. The database includes information on the procedural process of care and safety outcomes of TIs performed within the PICU. Pertinent clinical data such as age, patient category, and indications for intubation were prospectively collected at the time of TI, with secondary verification by research personnel and review of medical records. The data collection form also specifically included an assessment for potential difficult airway. In addition, data with regard to practice and provider factors were collected. These included intubation methods used (e.g., oral, nasal), choice of medications, training level (e.g., resident, fellow, attending) and discipline (anesthesia, critical care) of the provider. For this investigation, we analyzed data from July 2010 to March 2013.

### Study cohort

We included all initial TIs that took place in PICUs that were performed for the indication of acute respiratory failure and involved the use of direct laryngoscopy. We excluded tracheal tube replacements. TIs that involved other methods (e.g., indirect laryngoscopy, laryngeal mask and bronchoscopy) to visualize the airway were also excluded.

### Definitions

Three airway management events, ‘Encounter’, ‘Course’ and ‘Attempt’, were explicitly defined *a priori*, as described previously [4, 13–15]. Briefly, ‘Encounter’ was defined as one episode of completed advanced airway management intervention, including tracheal intubation. ‘Course’ was defined as one method or approach to secure an airway (e.g., oral vs. nasal, awake vs. sedated, standard vs. rapid sequence) and one set of medications including premedication and induction. An ‘Attempt’ was defined as a single advanced airway maneuver (e.g., beginning with the insertion of the device such as laryngoscope into patient’s mouth or nose, and ending when the device was removed). In the current study, the first course of each TI encounter was included for analysis. If the patient had more than one course before successful intubation (e.g., switch from direct laryngoscopy approach to laryngeal mask airway), then only the first course was included. We categorized the number of TI attempts into three groups: ‘one attempt’, ‘two attempts’, and ‘3 or more attempts’.

Our primary outcome was desaturation during TI. Desaturation was defined as the lowest SpO_2_ < 80 % during TI procedure in patients with SpO_2_ > 80 % after pre-oxygenation. We considered severe desaturation to be lowest SpO_2_ < 70 % in patients with SpO_2_ > 70 % after pre-oxygenation. Our secondary outcomes were adverse TI associated events (TIAEs). In the NEAR4KIDS, adverse TIAEs were prospectively categorized into two groups: severe and non-severe TIAEs [13]. Severe TIAEs included cardiac arrest with or without return of spontaneous circulation, esophageal intubation with delayed recognition, emesis with witnessed aspiration, hypotension requiring treatment, laryngospasm, malignant hyperthermia, dental trauma, and air leak (pneumothorax and/or pneumomediastinum). The following were considered as non-severe TIAEs: mainstem bronchial intubation, emesis without aspiration, hypertension requiring treatment, epistaxis, lip trauma, medical errors (not otherwise leading to severe TIAE), dysrhythmias, and pain and/or agitation requiring additional medication and causing delay in intubation.

### Statistical analysis

We summarized categorical variables as percentages and non-normally distributed continuous variables as medians and interquartile ranges. For univariate analysis, the chi-square test for categorical or dichotomous variables and the Wilcoxon rank-sum test for non-parametric variables were applied as appropriate. The number of TI attempts was categorized as ‘one attempt’, ‘two attempts’, and ‘three or more attempts’. Multivariate logistic regression was performed to evaluate the impact of the number of attempts on TI safety outcomes (desaturation, severe desaturation, occurrence of any TIAEs and severe TIAEs) while also adjusting for age, history of difficult airway, and upper airway obstruction. Age, history of difficult airway, and upper airway obstruction were identified as potential confounders because they were associated with the occurrence of TIAEs in previous studies [12, 14, 16]. We categorized patient age as infant (<1 year old), 1–7 years, and 8 years or older. We assessed the fit of the models using the Hosmer-Lemeshow test for adequate fit. We analyzed the data using STATA 11.2 (StataCorp, College Station, TX), with a two-sided *p-value* < 0.05 as the criterion for statistical significance. For sensitivity analyses, we excluded patients with cyanotic heart disease and repeated the analyses for desaturation and severe desaturation. We also separately completed a sensitivity analysis for desaturation and severe desaturation outcomes in the cohort of TIs with SpO2 ≥ 90 % after pre-oxygenation.

## Results

**Fig. 1:**
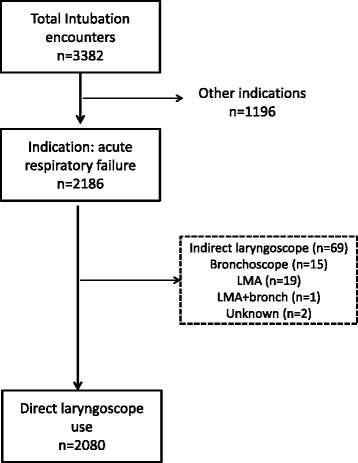
Study inclusion diagram

Of 3382 TIs, 2080 (65 %) were for acute respiratory failure and direct laryngoscopy was utilized (Fig. 1). The median age was 1 year (IQR: 0–5) and the median weight of the patients was 9.8 kg (IQR: 5–19.5), shown in Table 1. First attempt success was achieved in 1256/2080 (60 %), second attempt in 503/2080 (24 %), and with 3 or more attempts in 321/2080 (15 %). 377/2080 (18 %) had TI indications for upper airway obstruction. Overall, 304/2080 (15 %) patients had previous history of difficult airway.

**Table 1:** Patient characteristics categorized by the number of TI attempts (*n* = 2080)

	1 attempt (*n* = 1256)	2 attempts (*n* = 503)	≥ 3 attempts (*n* = 321)	*p*-value
Age in years median (IQR)	1 (1–6)	1 (0–6)	0 (0–4)	0.039
< 1 year	530 (42 %)	226 (45 %)	163 (51 %)	
1–7 years	459 (37 %)	173 (34 %)	99 (31 %)	
≥ 8 years	267 (21 %)	104 (21 %)	59 (18 %)	
Weight in kg median (IQR)	10 (5–20)	10 (5–19)	9 (4–18)	0.017
Male gender, n (%)	710 (57 %)	299 (60 %)	193 (61 %)	0.33
Patient category^a^				
Respiratory	685 (57 %)	294 (61 %)	168 (54 %)	
Cardiac	174 (15 %)	57 (12 %)	48 (16 %)	
Neurological	103 (9 %)	37 (8 %)	23 (7 %)	
Sepsis/Shock	59 (5 %)	25 (5 %)	23 (7 %)	
Trauma	18 (2 %)	7 (1 %)	10 (3 %)	
Other/Missing	153 (13 %)	64 (13 %)	37 (12 %)	
Indications^b^				
Oxygenation failure	736 (59 %)	299 (59 %)	183 (57 %)	0.79
Ventilation failure	723 (58 %)	304 (60 %)	206 (64 %)	0.08
Upper airway obstruction	225 (18 %)	96 (19 %)	56 (17 %)	0.80
History of difficult airway	168 (13 %)	75 (15 %)	61 (19 %)	0.04
Sign of upper airway obstruction^c^	153 (12 %)	74 (15 %)	64 (20 %)	0.001

*P*-value was calculated by Chi-square test

IQR denotes interquartile range

^a^Diagnostic category was missing in 95 encounters

^b^Each encounter may have more than one respiratory indication

^c^Physical sign was reported by providers at the time of tracheal intubation

A substantial proportion [863/2080 (42 %)] of first attempt providers were critical care fellows. The proportion of pediatric resident as the first attempt provider was significantly higher in TIs with multiple attempts (*p* < 0.001) (Table 2). The oral TI method was utilized in the majority of the TI attempts [2000/2080(96 %)]. Neuromuscular blockade was utilized in 1920/2080 (92 %) of the TI attempts. Use of neuromuscular blockade was not associated with the number of TI attempts (*p* = 0.85).

**Table 2:** Provider and practice factors categorized by the number of TI attempts (*n* = 2080)

	1 attempt (*n* = 1256)	2 attempts (*n* = 503)	≥ 3 attempts (*n* = 321)	*p*-value
1st attempt provider				
Pediatric Resident	161 (13 %)	164 (33 %)	123 (38 %)	<0.001
Critical Care Fellow	578 (46 %)	175 (35 %)	110 (34 %)	
Critical Care Attending	196 (16 %)	54 (11 %)	20 (6 %)	
Other/unspecified	321 (25 %)	110 (21 %)	68 (22 %)	
Method				
Oral	1196 (95 %)	488 (97 %)	316 (98 %)	0.01
Nasal	60 (5 %)	15 (3 %)	5 (2 %)	
Medication				
Vagolytic	503 (40 %)	206 (41 %)	162 (50 %)	0.003
Fentanyl	796 (63 %)	337 (67 %)	199 (62 %)	0.26
Midazolam	749 (60 %)	319 (63 %)	201 (63 %)	0.28
Ketamine	398 (32 %)	141 (28 %)	100 (31 %)	0.32
Propofol	89 (7 %)	29 (6 %)	11 (3 %)	0.047
Etomidate	15 (1 %)	5 (1 %)	4 (1 %)	0.93
Neuromuscular blockade	1156 (92 %)	466 (93 %)	298 (93 %)	0.85

Desaturation (SpO_2_ < 80 %) and severe desaturation (SpO_2_ < 70 %) were commonly observed in this study cohort (27 and 21 % of all TIs respectively). On univariate analysis, there was an increase in proportion of patients with desaturations (SpO_2_ < 80 %) with increasing number of TI attempts (Table 3). Compared to 202/1256 (16 %) that experienced desaturations in the ‘1 attempt’ group, 180/503 (36 %) and 179/321 (56 %) in the ‘2 attempts’ and ‘≥ 3 attempts’ groups respectively had desaturations (*p* < 0.001). Similarly, patients in the ‘2 attempts’ [147/504 (29 %)] and ‘≥3 attempts’ [122/321(38 %)] groups had more TIAEs compared to patients in the ‘1 attempt’ group [124/1256(10 %)] (*p* < 0.001). Hypotension requiring treatment occurred in 69/2080 (3 %) and was the most commonly reported severe TIAE (Table 4). The most common non-severe TIAE was esophageal intubation with immediate recognition [172/2080(8 %)].

**Table 3:** Univariate analysis for desaturation, severe desaturation, adverse tracheal intubation associated events (TIAEs) and severe TIAEs

	1 attempt (*n* = 1256)	2 attempts (*n* = 503)	≥ 3 attempts (*n* = 321)	*p*-value
Desaturations (<80 %)^a^	202 (16 %)	180 (36 %)	179 (56 %)	<0.001
Severe desaturations (<70 %)^b^	153 (12 %)	150 (30 %)	142 (44 %)	<0.001
Any TIAEs^c^	124 (10 %)	147 (29 %)	122 (38 %)	<0.001
Severe TIAEs	67 (5 %)	42 (8 %)	30 (9 %)	0.008

*P*-value was calculated by Chi-square test

Our sensitivity analysis limited to tracheal intubations (TIs) with pre-oxygenation SpO2 ≥ 90 % yielded a similar result (*N* = 1753): Desaturation (<80 %) was seen in 15 % of TIs with one attempt, 39 % in TIs with two attempts 59 % in TIs with three attempts (*p* < 0.001, Chi-square); severe desaturation (<70 %) was seen in 9 % of TIs with one attempt, 29 % in TIs with two attempts 44 % in TIs with three attempts (*p* < 0.001, Chi-square)

^a^Desaturation is defined as lowest pulse oximetry <80 % in patients with pulse oximetry >80 % after pre-oxygenation

^b^Severe desaturation is defined as lowest pulse oximetry <70 % in patients with pulse oximetry >70 % after pre-oxygenation

^c^TIAE denotes tracheal intubation associated events

**Table 4:** Description of tracheal intubation associated events

	1 attempt (*n* = 1256)	2 attempts (*n* = 503)	≥ 3 attempts (*n* = 321)
Severe TIAEs^a^			
Cardiac arrest with ROSC^b^	12 (1.0 %)	8 (1.6 %)	11 (3.4 %)
Cardiac arrest without ROSC	3 (0.2 %)	4 (0.8 %)	1 (0.3 %)
Esophageal intubation without immediate recognition	2 (0.2 %)	5 (1.0 %)	5 (1.6 %)
Emesis with aspiration	5 (0.4 %)	6 (1.2 %)	16 (0.8 %)
Hypotension requiring treatment	42 (3.3 %)	20 (4.0 %)	7 (2.2 %)
Laryngospasm	1 (0.1 %)	1 (0.2 %)	2 (0.6 %)
Malignant hyperthermia	0 (0.0 %)	0 (0.0 %)	0 (0.0 %)
Pneumothorax/pneumomediastinum	2 (0.2 %)	0 (0.0 %)	2 (0.6 %)
Dental trauma	1 (0.1 %)	2 (0.4 %)	0 (0.0 %)
Non-severe TIAEs			
Mainstem bronchial intubation	25 (2.0 %)	24 (4.8 %)	17 (5.3 %)
Esophageal intubation with immediate recognition	10 (0.8 %)	74 (14.7 %)	88 (27.4 %)
Emesis without aspiration	9 (0.7 %)	3 (0.6 %)	4 (1.3 %)
Dysrhythmia (includes sinus bradycardia)	10 (0.8 %)	15 (3.0 %)	9 (2.8 %)
Hypertension requiring treatment	1 (0.1 %)	2 (0.4 %)	0 (0.0 %)
Epistaxis	2 (0.2 %)	2 (0.4 %)	1 (0.3 %)
Lip trauma	1 (0.1 %)	3 (0.6 %)	5 (1.6 %)
Medication Error	1 (0.1 %)	0 (0.0 %)	1 (0.3 %)
Pain/Agitation requiring additional medication with delay in tracheal intubation	3 (0.2 %)	5 (1.0 %)	1 (0.3 %)

Note: If the patient had more than one course before successful intubation (e.g., switch from direct laryngoscopy approach to laryngeal mask airway), then only the first course was included for analysis. Therefore there were patients who had one attempt but still had an esophageal intubation

^a^TIAEs denotes tracheal intubation associated events

^b^ROSC denotes return of spontaneous circulation

After adjusting for patient factors (age, history of difficult airway, and upper airway obstruction), compared to ‘1 attempt’, the odds of developing desaturations increased with increasing number of attempts [‘2 attempts’, OR 2.9 (95 % CI: 2.3–3.7, *p* < 0.001) and ‘≥3 attempts’, OR 6.5 (95 % CI:5.0–8.5), *p* < 0.001)] (Table 5). The odds of adverse TIAE also increased with increasing number of attempts [‘2 attempts’, OR 3.7 (95 % CI: 2.9–4.9, *p* < 0.001) and ‘≥ 3 attempts’, OR (5.5, 95 % CI: 4.1–7.4), *p* < 0.001)]. The odds of severe TIAE also increased with increased number of attempts [‘2 attempts’, OR 1.6 (95 % CI: 1.1–2.4, *p* = 0.02) and ‘≥ 3 attempts’, (OR 1.8 (95 % CI: 1.1–2.8, *p* = 0.01)]. A sensitivity analysis for desaturation and severe desaturation excluding patients with cyanotic heart disease produced similar results (results not shown). Similarly a sensitivity analysis with a limited the cohort with TIs with SpO2 ≥ 90 % after pre-oxygenation yielded similar results (Table 3, footnote).

**Table 5:** Multivariate analysis for desaturation, severe desaturation, any TIAEs and severe TIAEs

	1 attempt (*n* = 1256)	2 attempts (*n* = 503)	≥ 3 attempts (*n* = 321)
Desaturation (SpO_2_ < 80 %)	1.0 (baseline)	2.9	6.5
(OR, 95 % CI, *p*-value)		[2.3–3.7, <0.001]	[5.0–8.5, <0.001]
Severe desaturation (SpO_2_ < 70 %)	1.0 (baseline)	3.1	5.7
(OR, 95 % CI, *p*-value)		[2.4–4.0, <0.001]	[4.3–7.5, <0.001]
Any TIAEs^a^	1.0 (baseline)	3.7	5.5
(OR, 95 % CI, *p*-value)		[2.9–4.9, <0.001]	[4.1–7.4, <0.001]
Severe TIAEs	1.0 (baseline)	1.6	1.8
(OR, 95 % CI, *p*-value)		[1.1–2.4, 0.02]	[1.1–2.8, 0.01]

Odds ratio, 95 % Confidence interval and p-value were calculated by multivariate logistic regression

Analysis adjusted for history of difficult airway, upper airway obstruction, age as a categorical variable (infant, 1–7 year, 8 or older). Note that history of difficult airway and signs of upper airway obstruction were identified as risk for multiple attempts [23]

Hosmer-Lemeshow goodness of fit test for each model: desaturation (SpO2 < 80 %): Chi2 (29) = 19.90, *p* = 0.90; severe desaturation (SpO2 < 70 %): Chi2 (29) = 20.69, *p* = 0.87; Any TIAEs: Chi2 (29) = 24.92, *p* = 0.68; Severe TIAEs: Chi2 (29) = 20.68, *p* = 0.87

For outcomes with any TIAEs, we ran a separate multiple logistic regression model without including esophageal intubation with immediate recognition as a part of TIAE definition. The result is shown in Additional file 2: Table S1

*OR* denotes odds ratio, *CI* denotes confidence interval

^a^TIAE denotes adverse Tracheal Intubation Associated Events

## Discussion

This multicenter study investigated the association between number of TI attempts and clinical outcomes in the PICU, accounting for patient characteristics previously associated with adverse events. We demonstrated that an increasing number of TI attempts in children with acute respiratory failure were associated with increased occurrence of desaturations, adverse TIAEs, and severe TIAEs. Desaturation (<80 %) was commonly observed in a quarter of the patients during TIs. The odds of desaturations increased approximately 3- and 6-fold with 2 attempts and ≥3 attempts of TIs, respectively. Adverse TIAEs were also observed in 19 % of patients undergoing TIs. The occurrences of any TIAE and severe TIAE were directly associated with increased number of attempts.

Our study adds strength to the current evidence in the medical literature that multiple TI attempts in critically ill children are associated with adverse clinical outcomes, even after accounting for patient characteristics such as age, history of difficult airway, and upper airway obstruction. In particular, our study focused on a unique cohort of children at high risk for desaturation during TIs. Patients with acute respiratory failure often have limited tolerance to apneic time (i.e., the time span without spontaneous ventilation, or positive pressure ventilation provided by an airway provider) which is required for the TI procedure. Therefore those children are perceived as particularly high risk for acute desaturation and physiological instability (hypotension, bradycardia) during a TI procedure. Our study was the first multi-center effort to quantify the impact of multiple attempts on this high risk pediatric population.

In critically ill adults, repeated TI attempts are associated with increased complications. In a seminal study of 2833 adults, ≥3 attempts in TI was associated with a 14-fold increased risk of severe desaturation (SpO_2_ < 70 %) and seven-fold increased risk for cardiac arrest [7]. Similar to these findings in adults, our findings add to the growing evidence that in critically ill children, multiple TI attempts are associated with worse clinical outcomes, including cardiac arrest (Table 4). In a previous single-center study of 137 TIs performed outside the operating room over a two-year period in a tertiary pediatric hospital, investigators reported complications in 56 (41 %) TI encounters [12]. Complications that were recorded in this study included desaturations, hypotension, bradycardia, vomiting and esophageal intubation. In that investigation, it was demonstrated that ≥3 attempts at intubation was an independent risk factor for complications (OR 2.3, 95 % CI: 1.3–4.3). Of note, the proportion requiring ≥3 attempts [20/137(15 %)] was similar to the proportion requiring ≥3 attempts [321/2080 (15 %)] in our investigation. While this study provided preliminary data on the risks associated with multiple TI attempts at a single center, our investigation of a large, multi-center cohort of patients (*n* = 2080) allowed us to precisely quantify the association of immediate clinical outcomes with number of TI attempts. In another prospective study conducted in a tertiary pediatric emergency department involving 71 TIs, >1 attempt was demonstrated to be associated with higher risk of adverse events (OR 7.7. 95 % CI: 2.0–26.5) [11]. In addition, a study that specifically examined TI in 105 pediatric trauma patients (with 151 TI attempts) reported that the risk of airway complication was 2.5-fold higher in children who required >1 attempt at TI [17]. The investigators demonstrated that multiple TIs were also associated with increased transport time, longer hospital length of stay and lower discharge Glasgow Coma Scale scores. However, not all studies involving TIs in children have reported increased risk with increasing TI attempts. A separate study that reported a similar proportion of TIs involving ≥3 attempts [36/281 (13 %)] did not demonstrate a significant association in the frequency of TIAEs between 1 attempt and >1 attempt groups [11/190 (6 %) vs. 10/91(11 %) respectively, *p* = 0.146]. This investigation was performed in a very different setting of a mixed adult-pediatric population in 13 emergency departments in Korea [10], which may suggest that different clinical areas with different spectrums of patient population may have an impact on the risk of TIAEs with increased number of attempts.

Another interesting finding in this investigation was the difference in the median age of patients across the three groups of TI attempts (Table 1). Patients that required ≥3 attempts were younger compared to the other two groups. This association between age of patient and number of TI attempts indicates that younger patients will be more likely to require multiple TI attempts. Our finding is congruent with findings from other studies that focused specifically on infants. In a study involving 203 infants in five tertiary neonatal intensive care units, investigators examined the characteristics of TIs over a one year period [18]. In contrast to our data where more than half of our patients required only 1 attempt, their study reported a higher number of attempts to establish a secure airway; 60/203 (30 %) required two attempts and 69/203 (34 %) required ≥3 attempts [18]. Unfortunately, this study did not report the incidence of desaturation or TIAEs with increasing number of TI attempts. Our findings in conjunction with the existing literature focusing on the younger age spectrum in pediatrics suggest that younger age is associated with higher risk of requiring multiple attempts in TIs and as such, higher risk for desaturation and adverse TIAEs.

In our study, pediatric residents were the first attempt provider more often in TIs requiring ≥3 attempts. This suggests the choice of first attempt provider may be an important modifiable factor to decrease number of TI attempts and related desaturation and adverse TIAEs [14, 16, 19]. To facilitate TI, sedation and neuromuscular blockade are often used. We did not find any association between the commonly used sedative drugs (e.g., fentanyl, midazolam) and number of TI attempts (Table 2). Neuromuscular blockade were used in a large majority [1920/2080 (92 %)] of the TI attempts in our study. We did not find any association between the use of neuromuscular blockade and number of TI attempts (Table 2). In an adult study involving 454 critically ill patients in two adult ICUs, the investigators reported a difference in the proportion of patients requiring “1 attempt” for successful TI with the use of neuromuscular blockade as compared to those without (85 % *vs.* 78 %, *p* = 0.047) [20]. Furthermore, this study demonstrated that neuromuscular blockade was associated with reduced risk of hypoxemia (OR 0.52, 95 % CI: 0.28–0.97) and complications (OR 0.29, 95 % CI: 0.11–0.78) during TI attempts.

Our study must be interpreted in the context of the limitations. Our primary aim focused on investigating the association between number of TI attempts and adverse clinical outcomes, accounting for 3 important patient characteristics previously associated with desaturation and adverse events: age, history of difficult airway, and upper airway obstruction. We acknowledge that there are other factors, in addition to TI attempts, that predispose a critically ill child to adverse clinical outcomes during the process of TI. We attempted to control for known confounders, however, we were not able to control for other important clinical covariates such as severity scores, as these data were not collected consistently across all sites in the database. We also recognize that the definition of desaturation and severe desaturation is somewhat arbitrary, although we established the definition in this investigation a priori based on previously published literature [7, 16, 21, 22]. Using these definitions, our primary analysis, along with a more restrictive sensitivity analysis, demonstrated a clear and strong association between number of attempts and desaturation. We also recognize the definitions for our secondary outcomes: adverse TIAEs and severe TIAEs were *a priori* developed by the NEAR4KIDS expert consensus, and each component may not have equal impact on patient outcomes. Therefore the results reported in this study are sensitive to the definitions. For example, when esophageal intubations with immediate recognition were removed from TIAE definition, the odds ratio for adverse TIAEs events attenuated from 3.7 to 1.6 (Table 5, Additional file 2: Table S1). Another limitation is the self-reported nature of the NEAR4KIDs database. There is a possibility of underreporting in the occurrence and degree of desaturation and adverse TIAEs, even though we attempted to limit this by ensuring complete capture of data with site specific compliance plans. Future studies with monitor waveform analysis may be able to address this issue by providing further detailed information regarding apneic time during TI attempts and effectiveness of rescue breaths after failed attempts. Another potential limitation is that the centers included in this database are largely academic medical centers and despite the multi-center, collaborative effort, the sites involved are not necessarily representative of all PICUs in North America or Asia Pacific. It is possible that generalizability of our findings to other PICUs may be somewhat limited.

Building on results provided by other investigators and our study team, we recognize the following points regarding TI in critically ill children: 1. Provider experience and status (e.g., residents, fellows and attendings) have an impact on first attempt and overall TI success [10, 11, 14]; 2. Risk of TIAEs is reduced with increasing experience of the initial provider [17]; 3. An increasing number of TI attempts is associated with increased risk of desaturations and TIAEs [10]; and 4. Children with difficult airways have a higher incidence of TIAEs during TI attempts [23]. Given these considerations, identification of which patients are safe and suitable for trainees to perform first TI attempt is of great importance to balance the need for training with patient safety. Currently several pediatric ICUs have implemented TI safety quality improvement bundle interventions to 1. Identify patients at risk for TIAEs and multiple attempts, 2. Generate a thoughtful airway management plan ahead of time, 3. Exercise a ‘timeout’ immediately before TI using a checklist, and 4. Conduct post TI procedure debriefing to identify strengths and room for improvement in technical skills and communication [24]. This ongoing quality improvement intervention aims to reduce adverse TIAE rates and multiple attempts. Cumulative evidence to date also sets the stage for future interventions (e.g., passive oxygen administration during TI attempts, [25] effective bag-mask ventilation for pre-oxygenation using real-time feedback system) to prevent severe desaturation when multiple TI attempts are required or anticipated.

## Conclusion

In summary, we demonstrated that an increasing number of TI attempts were independently associated with desaturation and adverse TIAEs in critically ill children with acute respiratory failure. Thoughtful selection of the initial intubating provider and optimizing intubating condition are important considerations to maximize the chance for first attempt success in order to optimize patient safety.

## Additional files

Additional file 1: List of investigators, participating sites with respective ethical boards’ approval. (DOCX 14 kb) Additional file 2: Table S1. Multivariate analysis for association between number of attempts and occurrence of any TIAEs without esophageal intubation with immediate recognition, and severe TIAEs without esophageal intubation with delayed recognition. (DOCX 15 kb)

## Abbreviations

CI: confidence interval
ICUs: intensive care units
IQR: interquartile range
MV: mechanical ventilation
NEAR4KIDS: National Emergency Airway Registry for Children
OR: odds ratio
PICU: pediatric intensive care units
SpO_2_: oxygen saturation on pulse oximetry
TI: tracheal intubation
TIAE: tracheal intubation associated event
TIAEs: tracheal intubation associated events
