# Supplementary material for: The number of tracheal intubation attempts matters! A prospective multi-institutional pediatric observational study
Source: BMC Pediatr. 2016 Apr 29;16:58. doi: 10.1186/s12887-016-0593-y (PMC4851769; doi:10.1186/s12887-016-0593-y)
Supplement: Additional file 1: — List of investigators, participating sites with respective ethical boards’ approval. (DOCX 14 kb) [file 12887_2016_593_MOESM1_ESM.docx]

**Appendix 1: List of investigators, participating sites with respective ethical boards’ approval.**

| **Investigators’ name** | **Participating ICU** |
| --- | --- |
| David. Turner, MD  Kyle J. Rehder, MD | Duke Children's Hospital, PICU |
| Michelle Adu-Darko, MD FAAP | University of Virginia Children’s Hospital, PICU |
| Sholeen Nett, MD PhD  J. Dean Jarvis, RN | Children’s Hospital at Dartmouth-Hitchcock Medical Center, PICU |
| Debbie Spear, RN | Penn State Hershey Children’s Hospital, PICU |
| Keith Meyer, MD FAAP | Miami Children’s Hospital, PICU |
| John S. Giuliano Jr, MD FAAP | Yale-New Haven Children’s Hospital, PICU |
| Vicki L Montgomery, MD FCCM | Kosair Children’s Hospital, PICU |
| Anthony Y. Lee, MD | Nationwide Children’s Hospital, PICU and CICU |
| Pradip Kamat, MD MBA | Children’s Healthcare of Atlanta – Emory University, PICU |
| Ronald C. Sanders Jr, MD MS | Arkansas Children’s Hospital, PICU |
| Simon Li, MD MPH  Matthew G. Pinto, MD FAAP | Maria Fareri Children’s Hospital at West Chester Medical Center, PICU |
| Keiko Tarquinio, MD | Hasbro Children’s Hospital, PICU |
| Joy D. Howell, MD FAAP FCCM | Children’s Hospital of New York Presbyterian Weill Cornell, PICU |
| Margaret M. Parker, MD MCCM | Stony Brook University Children’s Hospital, PICU |
| Jan Hau Lee, MBBS, MRCPCH, MCI | KK Women’s and Children’s Hospital, PICU |
| Gabrielle A. Nuthall, FRACP FCICM MBChB | Starship Children’s Hospital, PICU |
| Kris Bysani, MD FAAP FCCM | Medical City Children’s Hospital, PICU |
| Ana Lia Graciano, MD FAAP FCCM | Children’s Hospital Central California, PICU |
| Guillaume Emeriaud, MD PhD | CHU Sainte-Justine Université de Montréal, PICU |
| Akira Nishisaki, MD MSCE  Vinay M. Nadkarni, MD FCCM  Natalie Napolitano, MPH RRT-NPS FAARC | The Children’s Hospital of Philadelphia, PICU and CICU |

CICU: Cardiac intensive care unit; PICU: Pediatric intensive care unit
