# Supplementary material for: The number of tracheal intubation attempts matters! A prospective multi-institutional pediatric observational study
Source: BMC Pediatr. 2016 Apr 29;16:58. doi: 10.1186/s12887-016-0593-y (PMC4851769; doi:10.1186/s12887-016-0593-y)
Supplement: Additional file 2: Table S1. — Multivariate analysis for association between number of attempts and occurrence of any TIAEs without esophageal intubation with immediate recognition, and severe TIAEs without esophageal intubation with delayed recognition. (DOCX 15 kb) [file 12887_2016_593_MOESM2_ESM.docx]

**Additional file 2: Table S1. Multivariate analysis for association between number of attempts and occurrence of any TIAEs without esophageal intubation with immediate recognition, and severe TIAEs without esophageal intubation with delayed recognition**

|  | 1 attempt  (n = 1246) | 2 attempts  (n=429) | ≥ 3 attempts  (n= 233) |
| --- | --- | --- | --- |
| Any TIAEs^‡^  (OR, 95% CI, p-value) | 1.0 (baseline) | 1.6  [1.1-2.5, 0.02] | 1.3  [0.8-2.3, 0.31] |
| Severe TIAEs^†^  (OR, 95% CI, p-value) | 1.0 (baseline) | 1.5  [0.96-2.2, 0.08] | 1.5  [0.95-2.5, 0.08] |

^‡^ TIAEs denotes tracheal intubation associated events. Note this analysis does not include esophageal intubation with immediate recognition as part of any TIAEs.

^†^ Severe TIAEs denotes severe tracheal intubation associated events. Note this analysis does not include esophageal intubation with delayed recognition as part of severe TIAEs.
